# Progress in Medicine

**Published:** 1896-04-04

**Authors:** 


### Progress in Medicine.

### DISEASES OF CHILDREN.

Dr. Græme M. Hammond[1] has recorded a series of ten cases of multiple neuritis, occurring in the city of Bridgeport. The patients were all under five years of age, and most of them under two years. Eight of them were attacked with very similar symptoms during the months of June and August of last year, and the illnesses ran a very similar course. For these reasons Dr. Hammond is inclined to trace the outbreak to some common cause, probably of an infective nature. The lower limbs were the parts chiefly affected, there being complete motor paralysis and tenderness, which was most marked over the large nerve trunks. The paralysis tended to spread to the upper extremities, and one case proved fatal from involvement of the medullary centres. Treatment did not seem to have much effect on the course of the disease, which usually terminated in recovery in from three to four months.

In a lecture on "Tracheotomy and its After-Treatment," Mr. H. L. Lack[2] gives the indications for this operation and the manner of its execution, along with an account of the possible complications and how to avoid them. The most important signs of obstruction in the trachea and larynx are (1) stridor and (2) recession of the lower part of the chest wall during inspiration. If these are absent, then tracheotomy is not called for, and attention to this rule will prevent the operation being performed in cases of asthma, broncho-pneumonia, cerebral abscess, or cerebral compression, as has happened on different occasions.

The method of feeding infants introduced and carried out by M. Budin, of Paris, is the subject of a communication by Sir William O. Priestley.[3] M. Budin considers that, in the absence of breast feeding, the most efficient substitute is found in cow's milk prepared in a special manner, and unaccompanied by the addition of any other substance. His method of preparation is as follows: A bottle containing fresh cow's milk sufficient in quantity for one meal is placed in a bath of boiling water, and left there for forty minutes. It is then allowed to cool, and in the cooling process the rubber stopper with which the bottle is supplied sinks in automatically and completely shuts off the milk from any possible contamination with the air. The milk itself is not actually boiled, as the boiling point of milk is higher than that of water; but it is rendered practically sterile, and in addition it is so altered that the curd of the milk is separated into minute particles, which do not form large masses in the stomach or intestine. The milk thus treated is quite as palatable as pure fresh milk, and is administered without the addition of any water, which, in the opinion of M. Budin, is one of the causes of indigestion. Mr. C. W. Cathcart[4] has devised a simple form of milk steriliser, in which the supply necessary for one day may be prepared in about twenty minutes, and can be used as required without any risk of contamination from the air when laid aside. The apparatus is simple and cheap, can be heated in a pot such as is to be found in every kitchen, and can be thoroughly and easily cleaned. Dr. Edmund Cantley[5] has introduced a special milk for infants ("cream milk"), for which he claims certain advantages over the somewhat similar preparation employed in America by Dr. Rotch. The milk is supplied by the Aylesbury Dairy Company in air-tight bottles, at the rate of sixpence per pint. Dr. Jacobi,[6] of New York, reviews the methods and theories which are now current in connection with the subject of infant feeding. If artificial feeding has to be employed, he believes in the addition of cereals to cow's milk from the day of birth onwards.

In the Bradshaw lecture on "Infective and Tubercular Osteitis as Causes of Arthritis in Childhood," Mr. N. C. Macnamara[7] has summed up the present state of our knowledge of these affections. He recognises that in both the acute and the chronic forms the disease is dependent on the action of micro-organisms in the growing cancellous tissue at the ends of the diaphysis. From this as a starting point there may follow local changes, acute or chronic (tubercular) arthritis, &c., or general systemic infection, septicæmia, tubercular meningitis, &c. The treatment in the acute infective cases must be radical—namely, a free incision down to the bone over the diseased area, a free opening into the medullary cavity by means of a trephine, followed by irrigations of the parts with antiseptic lotions and drainage. In the chronic forms he does not consider that the old treatment by rest has given satisfactory results, and recommends that, as soon as a diagnosis has been made, an operation should be carried out similar to that mentioned above. In making the diagnosis in tubercular cases the exact site of the lesion is somewhat difficult to determine, but aid is afforded by the detection on careful examination of some persistently tender spots which indicate areas of tubercular osteitis, and must be thoroughly eradicated.

In cases of "Nocturnal Enuresis" Dr. Stumpf[8] finds that elevation of the pelvis by means of a pillow under the thighs will often check this troublesome disorder. His theory is that when a child occupies the ordinary position in sleep a few drops of urine find their way past the sphincter of the bladder into the urethra, and at once excite a powerful contraction of the bladder, whereas if the urine is allowed to gravitate backwards towards the fundus, this reflex action of the bladder is not excited.

In the treatment of whooping cough, Dr. G. Cavalieri[9] has obtained good results by means of vaccination. He vaccinated 100 children suffering from whooping cough, in 64 of whom well-marked vesicles appeared, and of these 64 only one died—a very small percentage compared with his previous mortality. He claims for vaccination in this connection that it shortens the duration of the disease, and diminishes the violence of the paroxysms. Dr. A. Rose[10] discusses the various methods adopted in the treatment of whooping cough, and concludes from his own experience that the best results are obtained by carbonic acid inflation of the rectum.

In a lecture on "School Surgery," Mr. A. M. Prichard[11] gives his experiences as surgeon to a large public school. The number of accidents at football is small considering the nature of the game, and the muscles suffer more frequently than the bones. Sprain of the flexor muscles of the thigh, and the rupture of some fibres of the quadriceps extensor, are not uncommon, and he has met with several cases of separation of the anterior inferior spine of the ileum by muscular action. Strain of the erector spinæ muscle is common, and is apt to recur. He considers that school runs do more good than harm, and in healthy boys seldom cause mischief. Cricket is noticed because of the general exhaustion and strain of the back which may follow fast and too prolonged bowling. For the younger boys at school he recommends a lighter ball and a shorter pitch than those used by adults. The medical aspects of school life are discussed by Dr. John Strachan,[12] who considers that teachers might with advantage proceed on more natural lines than they do at present.

The question as to whether rickets ever manifests itself for the first time in the later years of childhood, or only reappears at that period in those who have suffered from the disease in infancy, is still *sub judice*. Dr. Edmund Cantley[13] is inclined to support the latter view, and relates a case to illustrate his experience. A female child aged eleven years came under his care suffering from irregular pyrexia, muscular flabbiness and wasting, and the active epiphyseal enlargement of the long bones which is characteristic of rickets. Evidences of rickets had been present since she was four years old. Marked improvement was noted after four months' treatment. A case of congenital rickets is recorded by Dr. Victor Safford.[14] The patient, who was the child of Italian emigrants, was four years old when first seen, and had suffered from birth from weakness and twisted bones. The condition was evidently due to rickets, and the child made a good recovery under suitable diet and codliver oil.

The first number of a new journal of children's diseases, named *Pediatrics*, appeared on January 1st, and is to be published fortnightly. The contents are chiefly of a clinical nature, with numerous extracts from the current literature bearing on this subject. The owner, Dr. Dillon Brown, of New York, and the editor, Dr. George Carpenter, of London, have secured a large number of collaborators, both English and American, to assist in the special departments.

[1] N. Y. Med. Rec., Nov. 9th, 1895. [2] Clin. Journ., Feb. 5th, 1896. [3] Brit. Med. Journ., Dec. 7th, 1895, p. 1,401. [4] Brit. Med. Journ., Jan. 4th, 1896. [5] The Lancet, Jan. 11th, 1896. [6] Pediatrics, Jan. 1st, 1896. [7] The Lancet, Dec. 7th, 1895. [8] N. Y. Med. Rec., Nov. 23rd, 1895. [9] Ep. Brit. Med. Journ., Sep. 28th, 1895; and Gaz. d. Osped., June 1st, 1895. [10] N. Y. Med. Journ., Nov. 30th, 1895, p. 692. [11] Brist. Med. Chir. Journ., Dec., 1895. [12] Edinb. Med. Journ., Nov., 1895. [13] Brit. Med. Journ., Jan. 4th, 1896. [14] Pediatrics, Jan. 1st, 1896.

